# Assessing the temporal profile and liking of sugar‐free spicy dark chocolates with different concentrations of *Capsicum baccatum* pepper and rebaudioside A

**DOI:** 10.1002/jsfa.14327

**Published:** 2025-05-04

**Authors:** Gabriel Almeida Rodrigues Martins, Pedro Pio Campregher Augusto, Valdecir Luccas, Helena Maria André Bolini

**Affiliations:** ^1^ Food Engineering and Technology Department School of Food Engineering, University of Campinas (UNICAMP) São Paulo Brazil; ^2^ Cereal and Chocolate Research Centre (CEREAL CHOCOTEC), Institute of Food Technology (ITAL) São Paulo Brazil

**Keywords:** sugar‐free chocolate, sensory science, time‐intensity analysis, consumer acceptance, *Capsicum*, rebaudioside A

## Abstract

**Background:**

As consumers growingly demand healthier confectionery products, sugar‐free chocolates with added spices become interesting options to align with the permissible indulgence trend. This study explored the temporal sensory profiles and consumer acceptance of spicy sugar‐free dark chocolates formulated with varying levels of rebaudioside A in the stevia extract (500, 800, and 980 g kg^−1^) and dehydrated *Capsicum baccatum* pepper (2.0, 3.5, and 5.0 g kg^−1^).

**Results:**

Increasing the concentration of rebaudioside A enhanced the intensity of sweetness, while higher pepper concentrations resulted in increased spiciness intensity and duration. However, an exception was observed with the highest concentration of pepper (5.0 g kg^−1^) combined with 980 g kg^−1^ rebaudioside A, where a significant decrease in spiciness intensity and duration occurred. While the samples were generally well‐accepted, higher spiciness intensity and prolonged spiciness duration negatively impacted consumer acceptance.

**Conclusion:**

The findings suggest that higher sweetness intensities, induced by increased rebaudioside A concentrations, may modulate the perception of spiciness. Despite the negative impact of excessive spiciness on liking, the samples demonstrated good consumer acceptance overall, underscoring the potential of sugar‐free spicy dark chocolates in meeting health‐conscious consumer preferences while balancing sweetness and spiciness. © 2025 The Author(s). *Journal of the Science of Food and Agriculture* published by John Wiley & Sons Ltd on behalf of Society of Chemical Industry.

## INTRODUCTION

Health‐conscious consumers increasingly seek products combining indulgence and health benefits, particularly in traditionally unhealthy food categories like confectionery.^1^ Concerns over added sugar consumption, a key factor in the global obesity epidemic and related diseases, have driven international measures to limit sugar use such as taxes and front‐of‐pack labelling.^2^ In 2021, 537 million people had diabetes, a figure projected to rise to 643 million by 2030.^3^ Additionally, half of the global adult population is expected to be overweight or obese by 2030.^4^

The sugar‐free chocolate market aligns with the ‘permissible indulgence’ trend by addressing sucrose overconsumption while offering indulgent products. Sugar‐free chocolate formulations often contain low‐calorie bulking agents and non‐nutritive high‐intensity sweeteners to replace the bulking and sweetening properties of sucrose, respectively.^5^ Among the latter, *Stevia rebaudiana* extract is particularly popular due to its natural origin and perception as a safer, healthier clean‐label alternative to synthetic sweeteners like aspartame and sucralose.^6^ Stevia is 100–300 times sweeter than sucrose, and some studies suggest that it may have potential nutraceutical effects against diabetes, obesity, hypertension, dental caries, and some cancers.^7^ Among the more than 40 known steviol glycosides, rebaudioside A is commercially interesting due to its abundance (20–60 g kg^−1^ of dry matter) and favourable sensory properties. Compared to stevioside, rebaudioside A has a higher sweetening potency and a lower bitter aftertaste.^8^

The additions of dehydrated fruits, spices, and botanical extracts to chocolates is a strategy to enhance their health benefits while creating innovative sensory profiles.^9^ Chocolate, a dispersion of sugar, cocoa, and/or milk solids in a crystallized lipid matrix, is an excellent medium for bioactive ingredients due to its high fat content, which protects labile compounds from degradation and masks undesirable flavours.^10^ *Capsicum* spp. fruits (hot chili peppers) have been used as spices for millennia due to their piquancy, antioxidant and antimicrobial properties, primarily associated with capsaicin and dihydrocapsaicin.^11^ Capsaicinoids activate TRPV1 receptors in the mouth, mediating pain and thermal responses. Other compounds like carotenoids, phenolics, and vitamins C and E contribute to the potential health benefits and food preservation qualities of hot chili peppers.^12^

Despite such benefits, there is surprisingly little research on the sensory properties of chocolates with hot chili peppers, particularly sugar‐free varieties. Temporal sensory evaluation techniques, such as the time‐intensity (TI) analysis, are valuable tools for measuring how key sensory attributes of a food product evolve during oral processing. The TI method, which has been in use for over 50 years, involves trained panellists quantifying the intensity of specific sensory attributes over time using a computer interface.^13^ This method provides reliable, continuous, quantitative data and has been widely used to assess the dynamic sensory properties of high‐intensity sweeteners in sugar‐free and low‐sugar chocolates.^14^, ^15^, ^16^ Moreover, the multiple time‐intensity analysis (MTIA) emerged as an advanced form of TI data visualization that graphically represents the sensory dynamics of a sample by overlaying intensity curves of multiple attributes over time. This technique is feasible when test parameters are uniform across attributes.^17^ The MTIA approach indicates how the intensity of sensory characteristics in the same sample evolves throughout product consumption, providing a comprehensive view of multiple attributes simultaneously, even though data for each attribute is separately collected.^16^

Replacing or reducing sucrose is challenging because high‐intensity sweeteners rarely replicate the flavour profile of sucrose, especially in terms of sweetness intensity, duration, and residual bitterness. These sweeteners can also influence the perception of other flavours in the food matrix. In this context, correlating TI data with consumer acceptance through multivariate statistical analyses can help identify the temporal preference drivers in such products.^18^

This research aims to investigate the impact of different levels of rebaudioside A and dehydrated *Capsicum baccatum* on the temporal sensory profile and consumer acceptance of sugar‐free spicy dark chocolates and identify potential temporal preference drivers.

## MATERIAL AND METHODS

### Material

The following ingredients and additives, with respective suppliers, were used in the manufacture of samples: cocoa liquor NA700 and deodorized cocoa butter (Cargill, Ilhéus, Brazil); maltitol powder 150–300 MESH (Zhejiang Huakang Pharmaceutical Co. Ltd, Quzhou City, China); soy lecithin and polyglycerol polyricinoleate (PGPR) Mycelle PR 90 BD (Cargill); *C. baccatum* red pepper powder 30 000 SHU (Fuchs Gewürze do Brasil Ltd., Itupeva, Brazil); stevia extract 500 g kg^−1^ rebaudioside A, stevia extract 800 g kg^−1^ rebaudioside A and stevia extract 980 g kg^−1^ rebaudioside A (Shandong Haigen Biotechnology Co. Ltd, Qufu, China).

### Manufacture of samples

Samples were manufactured at the Centre for Chocolate and Cereals Technology (Institute of Food Technology, Campinas, Brazil), following the alternative processing method of conching ingredients prior to ball‐mill refining described in de Freitas *et al*.^19^ As shown in Table 1, maltitol was used as bulking agent and variables were the concentrations of rebaudioside A in the stevia extract (500, 800, and 980 g kg^−1^) and dehydrated pepper added to the chocolate mass (2.0, 3.5, or 5.0 g kg^−1^). Stevia concentration was based on its equivalent sweetness to sucrose in bittersweet chocolate described by Azevedo *et al*.,^20^ whereas the amount of dehydrated pepper was derived from the human threshold values for capsaicin^21^ and its average content in *C. baccatum*.^22^

**Table 1:** Formulations and coding of the spicy sugar‐free dark chocolate samples

Ingredients (g kg^−1^)	P20S50	P20S80	P20S98	P35S50	P35S80	P35S98	P50S50	P50S80	P50S98
Maltitol	445.0	445.0	445.0	445.0	445.0	445.0	445.0	445.0	445.0
Cocoa mass	440.0	440.0	440.0	440.0	440.0	440.0	440.0	440.0	440.0
Cocoa butter	110.0	110.0	110.0	110.0	110.0	110.0	110.0	110.0	110.0
Soy lecithin	3.0	3.0	3.0	3.0	3.0	3.0	3.0	3.0	3.0
PGPR	2.0	2.0	2.0	2.0	2.0	2.0	2.0	2.0	2.0
Pepper	2.0	2.0	2.0	3.5	3.5	3.5	5.0	5.0	5.0
Stevia 500 g kg^−1^ rebaudioside A	1.6	—	—	1.6	—	—	1.6	—	—
Stevia 800 g kg^−1^ rebaudioside A	—	1.6	—	—	1.6	—	—	1.6	—
Stevia 980 g kg^−1^ rebaudioside A	—	—	1.6	—	—	1.6	—	—	1.6

*Note*: PGPR, polyglycerol polyricinoleate.

Chocolate masses were manufactured in three distinct batches, each one containing different concentrations of pepper. Initially, maltitol and melted cocoa liquor were pre‐conched at 70 °C for 240 min in a 10‐kg rotary conching machine (Royal Duyvis Wiener Brazil, Tambaú, Brazil). Subsequently, melted cocoa butter was added and the mass was mixed for an additional 450 min at 60 °C. At the end, emulsifiers and pepper were added and the mass was let to liquefy for 30 min at 50 °C.

The three batches were then individually refined in a laboratory‐scale 5‐L ball mill, model CAO‐B5 (Caotech, Wormerveer, The Netherlands) until a maximum particle size of 20–25 μm, which took approximately 90 min at 75 Hz. Chocolates were kept at 50 ± 2 °C during the refining process by an external thermostatic bath. Maximum particle size was measured in triplicate with a digital micrometre (Mitutoyo Sul Americana, Suzano, Brazil), according to the method described by Luccas.^23^ The chocolates were stored in airtight 5‐L plastic containers at 20 °C until further use.

Each chocolate mass was melted and divided into three equal portions, with powdered stevia extracts with different rebaudioside A concentrations added just before tempering, resulting in nine distinct samples. Each sample was then heated to 45 °C and manually stirred on a marble slab until it reached 29 °C. The tempered chocolates were then moulded in 100‐g bars using polycarbonate moulds and cooled in an 8‐m cooling tunnel (Siaht, Jundiaí, Brazil). Samples were packed in aluminium foil and stored at 20 °C until further use.

### Sensory analysis

All sensory trials were conducted at the Laboratory of Sensory Science and Consumer Research, School of Food Engineering (University of Campinas, Campinas, Brazil) in individual booths equipped with computers and white lighting at a controlled temperature of 22 ± 1 °C. Ethical approval was obtained from University of Campinas' Research Ethics Committee (project number 40620820.1.0000.5404). Each participant received and signed an informed consent form detailing their involvement in the study.

#### Time‐intensity (TI) analysis

Thirty volunteers who consumed dark chocolate and did not reject spicy foods underwent preselection based on their discriminatory power. A series of triangle tests were conducted at a 1% significance level for sweetness, using two milk chocolate samples with different sucrose concentrations.^24^ Results were applied to Wald's sequential analysis and criteria for rejecting and accepting assessors were, respectively, thresholds of *P* = 0.45 for the maximum acceptable inability and *P*_1_ = 0.70 for the minimum acceptable ability to discriminate between samples. Moreover, risk parameters were set at *α* = 0.05 for the probability of approving an assessor lacking discriminatory power and *β* = 0.05 for the probability of rejecting an assessor with suitable discriminatory power.^25^

Twenty‐one preselected assessors participated in six 60‐min training sessions with the physical references of maximum intensities of sweetness, bitterness, and spiciness described in Table 2. After completing training, assessors evaluated samples using the Time‐Intensity Analysis of Flavors and Tastes (TIAFT) software,^26^ according to the following setup: (i) a 5‐s waiting period; (ii) 15 s of in‐mouth residence time; (iii) post‐ingestion evaluation periods of 90 s for sweetness, 120 s for bitterness, and 180 s for spiciness; and (iv) a structured linear scale ranging from zero (none) to ten (strong). Throughout evaluation, the software simultaneously provided subtle sound signals and on‐screen alerts to inform the assessors of the predefined time intervals. The intensity of each attribute over time was recorded by moving a cursor along the scale.^27^ Samples, presented as 1 × 1 cm^2^ squares, were evaluated for each one of the attributes in separate sessions, in triplicate, and following a sequential monadic order within a complete block balanced design.^28^

**Table 2:** Definitions and physical references of maximum intensity of stimuli used in panel training for time‐intensity analysis

Attribute	Definition	References
Sweetness	Taste associated with the presence of sucrose or other sweeteners	Solution of 2.0 g kg^−1^ stevia with 980 g kg^−1^ rebaudioside A
Bitterness	Bitter taste of cocoa solids in dark chocolates	Mixture of 320 g kg^−1^ melted cocoa butter, 300 g kg^−1^ maltitol and 280 g kg^−1^ cocoa powder
Spiciness	Burning sensation caused by capsaicinoids in mouth	Aqueous solution of 15 g kg^−1^ capsaicin in a 20 g kg^−1^ cocoa butter water‐in‐oil emulsion

After evaluation, the following parameters were obtained from the TI curves: (i) maximum intensity of stimulus (*I*_max_); (ii) time at which perception of stimulus started (*T*_onset_); (iii) time to reach maximum intensity (*T*_max_); (iv) total duration of stimulus (*T*_tot_); and (v) the area under the curve (AUC). The extraction of *I*_max_, *T*_max_, *T*_tot_, and AUC from the TI curves follows established methodologies in sensory analysis. These parameters have been widely used to describe the temporal dynamics of perception, as they provide key insights into peak intensity, duration, and overall impact of sensory attributes.^29^

For the assessor validation, data of the TI curve parameter were analysed individually for each assessor using a two‐way analysis of variance (ANOVA), with sample (pF_sam_) and repetition (pF_rep_) as sources of variation, using the Statistical Analysis System (SAS) software, version 9.4.^30^ Those who demonstrated suitable discriminative ability between samples (pF_sam_ ≤ 0.50) and reproducibility within repetitions (pF_rep_ ≥ 0.05) for all the parameters of the curve were selected to compose the final trained panel.^31^ The number of selected assessors varied by attribute: 15 for sweetness, 13 for bitterness, and 14 for spiciness. The TI data from the qualified assessors were subsequently processed and analysed using statistical methods described in a separate section.

#### Consumer acceptance test

The acceptance test was conducted in two separate sessions to minimize sensory fatigue due to the spiciness of the pepper. Subjects were mainly undergraduate students at the University of Campinas, recruited via social media. They were chosen based on availability, familiarity with dark chocolate and no aversion to chili peppers. A total of 120 subjects evaluated the samples monadically, rating appearance, aroma, flavour, texture, and overall impression on a 9‐cm unstructured scale ranging from ‘dislike extremely’ to ‘like extremely’ using FIZZ Sensory Analysis Software (Biosystèmes, Courtenon, France).^32^

#### Statistical data processing

TI data from qualified assessors were statistically analysed in SAS software using two‐way ANOVA, with sources of variation being samples, assessors, and sample × assessor interactions, followed by Tukey's honestly significant difference (HSD) test at a 5% significance level. Furthermore, the means of maximum intensity (*I*_max_), initial time of perception (*T*_onset_), time to maximum intensity (*T*_max_), and total duration (*T*_tot_) of each attribute were simultaneously plotted in MTIA graphs.^17^

Consumer acceptance data were analysed in SAS software by two‐way ANOVA (subject and sample) and Tukey's HSD test (*P < 0.05*). To investigate potential preference drivers among the temporal sensory attributes, partial least squares regression (PLSR) analysis was conducted in XLSTAT software for Windows version 2024.3.0 (Addinsoft, Paris, France), using the TI parameters’ means as explanatory variables and overall impression means from consumer acceptance as dependent variable. The PLSR model quality was assessed using *Q* ^2^ cumulative (0.140), *R* ^2^ *Y* cumulative (0.570), and *R* ^2^ *X* cumulative (0.389). The goodness‐of‐fit statistics for overall impression showed an *R* ^2^ of 0.570, root mean squared error of 0.151, and mean squared error of 0.023, suggesting a moderate explanatory power.^33^

## RESULTS AND DISCUSSION

### Time‐intensity (TI) analysis

#### Sweetness

Table 3 shows the results of ANOVA and Tukey's HSD test for the sweetness’ TI curve parameters. As expected, the concentration of rebaudioside A exerted a major effect on *I*_max_ values, which were significantly higher (*P* ≤ 0.05) in samples containing 980 g kg^−1^ rebaudioside A than in those with 500 and 800 g kg^−1^. An exception is sample P20S80, which did not significantly differ (*P* > 0.05) from samples P20S98 and P50S98. An extra glucosyl moiety linked at the C‐13 position of the steviol structure accounts for the increased sweetness of rebaudioside A in comparison to stevioside.^34^

**Table 3:** Results of Tukey's HSD (honestly significant difference) test for the time‐intensity parameters of sweetness

Sample	*I*_max_	*T*_onset_ (s)	*T*_max_ (s)	*T*_tot_ (s)	AUC
P20S50	6.554^d^	8.269^a^	20.687^ab^	72.014^ab^	334.35^ab^
P20S80	7.163^bc^	8.536^a^	19.908^b^	72.012^abc^	326.27^b^
P20S98	7.667^ab^	8.906^a^	22.263^ab^	79.507^a^	363.11^ab^
P35S50	6.537^d^	9.301^a^	21.796^ab^	72.508^abc^	316.51^b^
P35S80	6.985^cd^	9.271^a^	22.472^ab^	74.249^abc^	343.58^ab^
P35S98	7.779^a^	8.605^a^	24.150^a^	79.346^ab^	384.51^a^
P50S50	6.484^d^	9.570^a^	22.139^ab^	67.131^c^	314.00^b^
P50S80	7.060^cd^	10.098^a^	21.150^ab^	71.504^bc^	317.81^b^
P50S98	7.735^ab^	8.755^a^	24.138^a^	78.644^ab^	360.19^ab^

*Note*: Means with the same superscript letters in the same column have no statistical difference at a 5% significance level.

*I*_max_, maximum intensity; *T*_onset_, initial time of perception; *T*_max_, time to maximum intensity; *T*_tot_, total duration; AUC, area under the curve.

Although *T*_onset_ showed no significant difference (*P > 0.05*) among samples, those with 980 g kg^−1^ rebaudioside A presented the highest *T*_max_ and *T*_tot_. Significant differences in *T*_max_ (*P* ≤ 0.05) were observed for samples P35S98 and P50S98 in relation to P20S80, and in *T*_*tot*_ for sample P20S98 in relation to samples P50S50 and P50S80. Moreover, 980 g kg^−1^ rebaudioside A samples presented higher AUC than those with either 500 g kg^−1^ or 800 g kg^−1^ rebaudioside A, as sample P35S98 significantly differed (*P* ≤ 0.05) from samples P20S80, P35S50, P50S50, and P50S80. Contrastingly, Azevedo *et al*.^14^ observed no significant difference (*P* > 0.05) in the temporal sweetness profile of bittersweet chocolates sweetened with either sucrose or 1.6 g kg^−1^ stevia at three different concentrations of rebaudioside A (600, 800, and 970 g kg^−1^). The authors hypothesized that the pronounced cocoa flavour, characteristic to bittersweet chocolates, could have affected sweetness perception, thus resulting in no significant differences between samples with different rebaudioside A concentrations.

#### Bitterness

Bitter taste is a common sensory characteristic in dark chocolates, caused by naturally occurring compounds in cocoa beans, that is, alkaloids and polyphenols, and those formed by protein degradation during roasting.^35^ Therefore, bitterness was expected in all samples, due to their relatively high cocoa mass content (440 g kg^−1^). Additionally, a bitter aftertaste was also anticipated in samples due to the stevia extracts.

The TI results for bitterness, shown in Table 4, suggest that higher concentrations of rebaudioside A accounted for an overall decrease in *I*_*max*_. This reduction was only statistically significant (*P* ≤ 0.05) between samples P20S98 and P50S80, though. Contrastingly, Azevedo *et al*.^14^ observed no significant differences in any of the TI parameters for bitterness among bittersweet chocolates sweetened with different levels of rebaudioside A.

**Table 4:** Results of Tukey's HSD (honestly significant difference) test for the time‐intensity parameters of bitterness

Sample	*I*_max_	*T*_onset_ (s)	*T*_max_ (s)	*T*_tot_ (s)	AUC
P20S50	5.52^ab^	12.51^a^	31.29^a^	78.31^a^	278.15^ab^
P20S80	5.26^ab^	12.27^a^	35.23^a^	78.56^a^	259.94^b^
P20S98	5.11^b^	12.26^a^	33.96^a^	79.83^a^	288.57^ab^
P35S50	6.08^ab^	13.13^a^	31.01^a^	84.94^a^	342.32^ab^
P35S80	5.88^ab^	12.69^a^	34.96^a^	78.46^a^	294.97^ab^
P35S98	5.61^ab^	13.25^a^	33.35^a^	76.42^a^	320.00^ab^
P50S50	5.78^ab^	13.22^a^	30.84^a^	84.12^a^	313.24^ab^
P50S80	6.21^a^	12.25^a^	32.62^a^	75.39^a^	312.02^ab^
P50S98	6.15^ab^	12.88^a^	34.76^a^	83.59^a^	357.00^a^

*Note*: Means with the same superscript letters in the same column have no statistical difference at a 5% significance level.

*I*_max_, maximum intensity; *T*_onset_, initial time of perception; *T*_max_, time to maximum intensity; *T*_tot_, total duration; AUC, area under the curve.

The intensities of sweetness and bitter aftertaste of steviol glycosides are positively and negatively correlated to the number of glucosyl moieties linked at the C‐13 and C‐19 positions, respectively. Although rebaudioside A shares the same number of glucose units at C‐19 as stevioside, it elicits a milder bitter aftertaste than the latter, most likely due to the extra glucose unit linked at C‐13.^36^ The suppression of bitterness by an increase in sweetness could be an instance of two gustatory stimuli being perceived concomitantly and influencing the intensity of one another.^20^

Overall, *I*_max_ rose as the concentration of pepper in samples increased. Literature lacks information on specific compounds that could be responsible for the bitter taste of peppers from the *Capsicum* genus. Some studies suggest that either aglycones or quercetins could elicit bitterness in *Capsicum annuum* sweet bell peppers.^37^, ^38^ However, capsaicin itself can stimulate bitter responses in some individuals by triggering TAS2R receptors,^39^ especially in the circumvallate region of the tongue, which could account for the higher bitterness *I*_max_ of samples with more pepper.

Results of *T*_onset_, *T*_max_, or *T*_tot_ did not show significant differences between samples. Surprisingly, the highest AUC was observed in a sample with 980 g kg^−1^ rebaudioside A, P50S98, which was significantly different (*P* ≤ 0.05) from sample P20S80. This could be due to the potential gustatory stimulation of capsaicin (5.0 g kg^−1^) or the interaction with other bitter tasting compounds in the complex multicomponent matrix of dark chocolate, and not a reflex of the rebaudioside A concentration itself.

#### Spiciness

Capsaicinoids are a class of hydrophobic vanilloid compounds that give peppers from the genus *Capsicum* their characteristic spiciness. They bind to TRPV1 receptors at the peripheral terminals of primary afferent neurons, triggering nociceptive and thermal stimuli.^40^ Krajewska and Powers^41^ observed a linear relationship between the concentration of isolated capsaicinoids and the magnitude of the perceived pungent sensation within the range of 0.039 to 0.625 mg kg^−1^. Capsaicin threshold concentrations are usually higher in oil than in aqueous solutions, likely because its lipophilic nature makes it interact more to lipidic matrices, which reduces its availability to trigger a chemesthetic response.^42^

Table 5 displays the results of the TI analysis for spiciness. As expected, both *I*_max_ and AUC increased with the rise in pepper concentration from 2.0 to 5.0 g kg^−1^, with statistically significant differences (*P* ≤ 0.05) observed between samples with contrasting pepper contents. A noteworthy exception was sample P50S98, which exhibited a significantly lower *I*_max_ (*P* ≤ 0.05) than other samples with the same pepper concentration and no significant difference (*P* > 0.05) from samples P20S50 and P20S80, which contained only 2.0 g kg^−1^ pepper.

**Table 5:** Results of Tukey's HSD (honestly significant difference) test for the time‐intensity parameters of spiciness

Sample	*I*_max_	*T*_onset_ (s)	*T*_max_ (s)	*T*_tot_ (s)	AUC
P20S50	4.42^e^	17.80^ab^	38.10^a^	79.58^de^	168.87^d^
P20S80	4.19^ef^	16.82^abc^	41.82^a^	78.36^e^	189.25^cd^
P20S98	3.11^f^	17.99^a^	37.81^a^	75.15^e^	151.85^d^
P35S50	6.35^bc^	16.44^abc^	36.89^a^	98.29^bc^	352.12^b^
P35S80	6.14^cd^	16.67^abc^	41.53^a^	99.94^bc^	361.63^b^
P35S98	5.57^cd^	16.25^abc^	36.75^a^	103.84^bc^	341.83^b^
P50S50	7.24^ab^	14.79^c^	38.01^a^	113.96^ab^	484.22^a^
P50S80	7.51^a^	15.54^bc^	41.66^a^	123.90^a^	525.78^a^
P50S98	5.25^de^	16.21^abc^	37.82^a^	96.35^cd^	285.46^bc^

*Note*: Means with the same superscript letters in the same column have no statistical difference at a 5% significance level.

*I*_max_, maximum intensity; *T*_onset_, initial time of perception; *T*_max_, time to maximum intensity; *T*_tot_, total duration; AUC, area under the curve.

Psychophysical studies suggest that primary tastes can influence the perception of capsaicin in the oral cavity, with sucrose solutions effectively suppressing the burning sensation induced by capsaicinoids.^40^ Additionally, Smutzer *et al*.^21^ observed a masking effect of oral rinses of isointense solutions of sucrose (175 mmol L^−1^) and sucralose (2.64 mmol L^−1^) upon the intensity of pungency provided by capsaicin‐loaded edible strips (5 nmol) by 50%. This implies either an interaction between taste receptor cells and trigeminal neurons occurring in the oral cavity or a signal integration of taste and chemesthetic stimuli taking place at the central nervous system.^40^ Results of TI analysis suggest that the increased sweetness intensity could have masked the perception of pepper's spiciness in the sample with maximum concentrations of both pepper (5.0 g kg^−1^) and rebaudioside A (980 g kg^−1^).

No significant differences (*P* > 0.05) were observed for *T*_max_, meaning that investigated variables did not significantly affect the time required for spiciness intensity to peak. However, *T*_tot_ varied significantly (*P* ≤ 0.05) between samples, as spiciness lasted longer in those with higher pepper levels. An exception was sample P50S98, whose total time of spiciness perception was significantly shorter than the other samples with 5.0 g kg^−1^ pepper. Moreover, spiciness lasted longer than sweetness, particularly in sample P50S80, in which it persisted for over 2 min. Schneider *et al*.^43^ found a low correlation between the total capsaicinoid content with spiciness *T*_tot_ in different spicy foods (*R* ^2^ = 0.518), despite showing a good correlation with spiciness *I*_max_ (*R* ^2^ = 0.803). This could be due to variations in matrix composition, especially in fat content, as capsaicinoids' lipophilic nature could delay their release from the matrix.

Moreover, it was interesting to note that the *T*_onset_ occurred mostly during post‐ingestion time (longer than 15 s), that is, after samples had fully melted and been ingested. There was no significant difference (*P* > 0.05) for *T*_max_ between samples, but it is noteworthy that it occurred after sweetness *T*_max_. Sinesio *et al*.^44^ explain that such delay can be attributed to the distinct afferent neural pathways transmitting either chemesthetic or gustative signals, as the former travel at a slower speed (2–30 m s^−1^) than the former (up to 100 m s^−1^), resulting in a distinct kinetic perception for these stimuli.

### Multiple time‐intensity analysis (MTIA)

**Figure 1:**
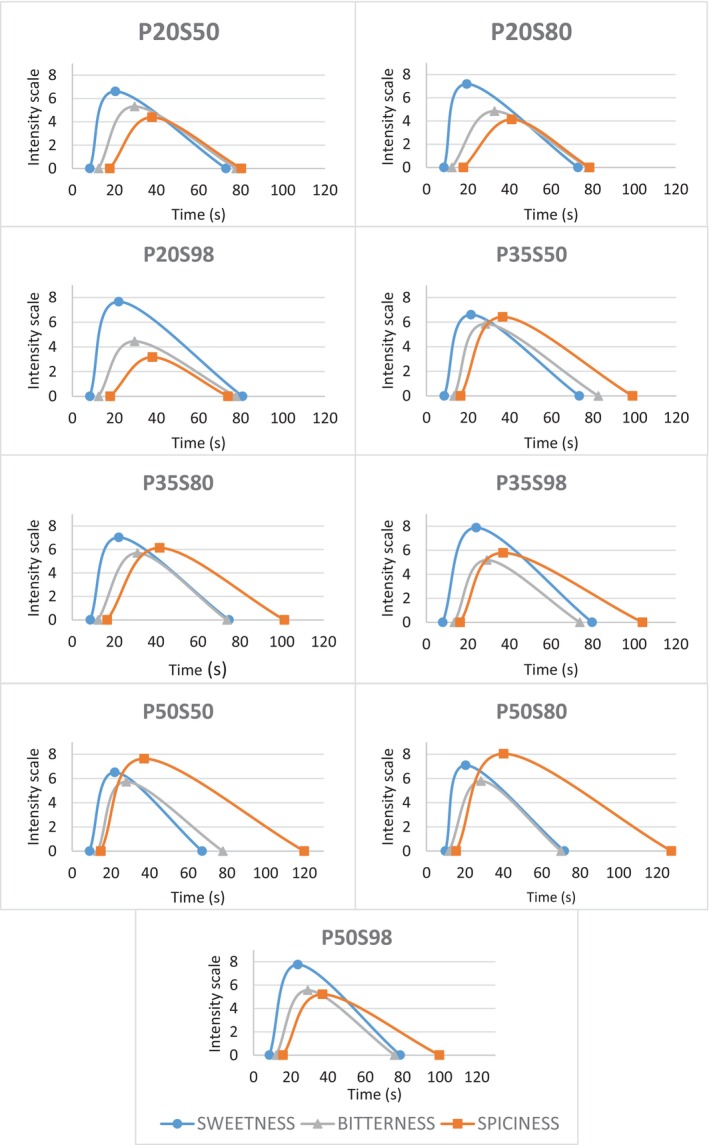
Multiple time‐intensity analysis (MTIA) of dark chocolate samples with either 2.0 g kg^−1^, 3.5 g kg^−1^, or 5.0 g kg^−1^ pepper (P20, P35, P50) and either 500 g kg^−1^, 800 g kg^−1^, or 980 g kg^−1^ rebaudioside A (S50, S80, S98) for sweetness, bitterness, and spiciness.

Figure 1 shows the MTIA curves of intensity *versus* time (in seconds) for each sample. It is possible to note that the gap between sweetness and bitterness curves widened as the concentration of rebaudioside A in the samples increased, due to the enhanced flavour profile of rebaudioside A in comparison to other steviol glycosides, that is, stevioside. Furthermore, the curve of spiciness intensity grew taller and wider, indicating an increase in *I*_max_, AUC, and *T*_tot_ values of spiciness as the concentration of dehydrated pepper in samples rose. An exception to this trend was sample P50S98, which was perceived as sweeter and much less spicy than the other samples with 5.0 g kg^−1^ pepper.

### Consumer acceptance test

Results of consumer acceptance test are presented in Table 6. Samples were generally well‐accepted by consumers, as they received ratings of seven or higher for overall impression. No significant difference (*P* > 0.05) was found for the acceptance of aroma. In contrast, liking of appearance showed a significant difference (*P* ≤ 0.05) between samples, and samples P20S80 and P50S80 scored the highest and lowest means, respectively. Interestingly, a statistically significant difference (*P* ≤ 0.05) for flavour also occurred between these samples, as the sample with 2.0 g kg^−1^ pepper received the highest score and the one with 5.0 g kg^−1^ pepper the lowest, at the same concentration of 800 g kg^−1^ rebaudioside A. Furthermore, the average of texture liking for sample P20S80 was significantly higher (*P* ≤ 0.05) than samples P35S80 and P35S98. Sample P20S80 was the most preferred overall, earning the highest ratings for aroma, flavour, texture, and overall impression.

**Table 6:** Results of Tukey's HSD (honestly significant difference) test for the consumer acceptance of appearance, aroma, flavour, texture and overall impression

Sample	Appearance	Aroma	Flavour	Texture	Overall impression
P20S50	7.478^ab^	7.556^a^	7.208^ab^	7.442^ab^	7.551^ab^
P20S80	7.653^a^	7.586^a^	7.426^a^	7.537^a^	7.678^a^
P20S98	7.476^ab^	7.457^a^	7.182^ab^	7.314^ab^	7.340^abc^
P35S50	7.435^ab^	7.361^a^	7.128^ab^	7.246^ab^	7.435^abc^
P35S80	7.330^abc^	7.388^a^	6.942^ab^	6.815^b^	7.249^abc^
P35S98	7.028^bc^	7.229^a^	6.944^ab^	6.827^b^	6.970^c^
P50S50	7.403^abc^	7.39^a^	7.290^ab^	7.077^ab^	7.137^abc^
P50S80	6.928^c^	7.247^a^	6.764^b^	6.919^ab^	7.034^bc^
P50S98	7.549^a^	7.472^a^	7.129^ab^	6.890^ab^	7.523^ab^

Means with the same superscript letters in the same column have no statistical difference at a 5% significance level.

In general, pepper concentration had a greater impact upon flavour and overall impression ratings than rebaudioside A concentration, as samples with the lowest pepper level were generally more well‐accepted than those with 3.5 g kg^−1^ or 5.0 g kg^−1^ pepper. Contrastingly, sample P50S98 did not significantly differ (*P* > 0.05) from sample P20S80, despite having the highest concentration of pepper. Its unexpectedly high overall impression rating could be attributed to the masking effect of the increased concentration of rebaudioside A upon the spiciness of the pepper, as observed in the MTIA.

### Preference drivers

**Figure 2:**
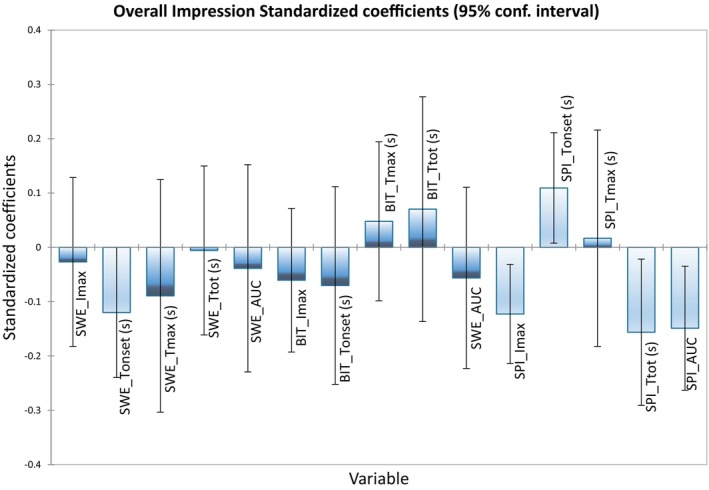
Results of the partial least squares regression (PLSR) between means of the time intensity parameters for sweetness (SWE), bitterness (BIT) and spiciness (SPI), and overall impression ratings, at 5% significance. Light‐blue boxes represent that there is either a negative or positive influence of the attribute upon consumer acceptance. *I*_max_, maximum intensity; *T*_onset_, initial time of perception; *T*_max_, time to maximum intensity; *T*_tot_, total duration; AUC, area under the curve.

Figure 2 shows which parameters of the TI curves negatively or positively influence consumer acceptance of the chocolates within the studied sample set, at a 5% significance level. Most temporal sensory attributes that affected liking were those related to the perception of spiciness, as *T*_onset_ contributed positively to consumer acceptance, whereas *I*_max_, *T*_tot_, and AUC showed a negative contribution (*P* ≤ 0.05). In other words, the longer and the more intensely spiciness was perceived in chocolates by consumers, the lower the acceptance ratings of samples were. Even though sweetness *T*_onset_ values did not vary significantly (*P* > 0.05) between samples, longer onset times for sweetness negatively impacted consumer liking.

## CONCLUSION

This study pioneered the investigation of the sensory properties of spicy sugar‐free dark chocolates and the impact of varying concentrations of *C. baccatum* pepper and rebaudioside A on their temporal profile and acceptance by consumers. Results showed that these chocolates were generally well‐received, underscoring the feasibility of incorporating spicy ingredients into sugar‐free dark chocolates. However, as the intensity and duration of the spicy stimulus increased, liking declined, which suggests that while the chocolate–pepper combination is appealing, excessive spiciness can overshadow its overall liking. Moreover, this study was the first to demonstrate a potential masking effect of rebaudioside A upon the perception of capsaicinoids' spiciness in a complex food matrix, a phenomenon previously reported for sucrose and sucralose in aqueous solutions.^21^ These findings highlight the critical role of balancing spiciness and sweetness to optimize consumer satisfaction in spicy sugar‐free chocolate formulations by means of adjusting the concentration of dehydrated pepper and selecting stevia extracts with appropriate rebaudioside A levels. Future research should explore individual differences in consumer preferences and investigate opportunities to tailor these products for specific market segments.

## CONFLICT OF INTEREST STATEMENT

The authors have no conflict of interest to declare.

## Data Availability

The data that support the findings of this study are available from the authors upon reasonable request.
